# Association and risk factors analysis of FeNO and CRP in bronchial asthma combined with obstructive sleep apnea

**DOI:** 10.3389/fmed.2025.1546389

**Published:** 2025-06-30

**Authors:** Zhao Yan, Wang Peng Fei, Wang Nai Zhu

**Affiliations:** ^1^Department of Respiratory and Critical Care Medicine, Affiliated Hospital of Hebei University of Engineering, Handan, Hebei, China; ^2^Department of Neurosurgery, Affiliated Hospital of Hebei University of Engineering, Handan, Hebei, China

**Keywords:** obstructive sleep apnea, bronchial asthma, fractional exhaled, nitric oxide, inflammatory markers

## Abstract

Bronchial asthma (BA) and obstructive sleep apnea (OSA) are chronic disorders of the respiratory system; both diseases are widespread and can cause a decrease in the quality of life. The latter contrasts the airway inflammation and excessive reactivity that define BA, and the intermittent airway obstruction during sleep that defines OSA results in periods of hypoxemia and disruptive breathing. BA appears when children have OSA in addition to other disorders, including night ventilation, breathlessness, and sleep conflicts. Current studies have focused on inflammatory indicators such as the FeNO and CRP in these diseases. FeNO is a measure of eosinophilic airway inflammation, which is usually high in asthma. In, contrast, CRP is a measure of systemic inflammation that is usually high in both forms of asthma. This review paper will focus on FeNO and CRP in connection to the pathophysiology of BA and OSA with further descriptions of how these markers relate to inflammation in both disorders. The review also focuses on how these markers interlink in patients with both diseases and how FeNO and CRP can also reflect the severity of the disease and the effectiveness of the treatments being used. Finally, identifying the interaction between these markers might advance the identification and management of patients with both BA and OSA.

## Introduction

Bronchial asthma (BA) and obstructive sleep apnea (OSA) are two widespread respiratory illnesses affecting most people worldwide (1). Although the two conditions are usually treated separately, their combined presence in the same patient has become a concern following the difficulties that arise when diagnosing and treating the two. BA combined with OSA was defined as the presence of asthma and OSA. The incidence of both these conditions has been rising in day-to-day practice, especially in patients who have common predisposing factors like obesity, aging, and other comorbidities of the respiratory system (2). Thus, as research on this relationship expands, awareness of the relationship between the two conditions, in the context of inflammatory markers such as fractional exhaled nitric oxide (FeNO) and C-reactive protein (CRP), has emerged (3).

**Figure 1:**
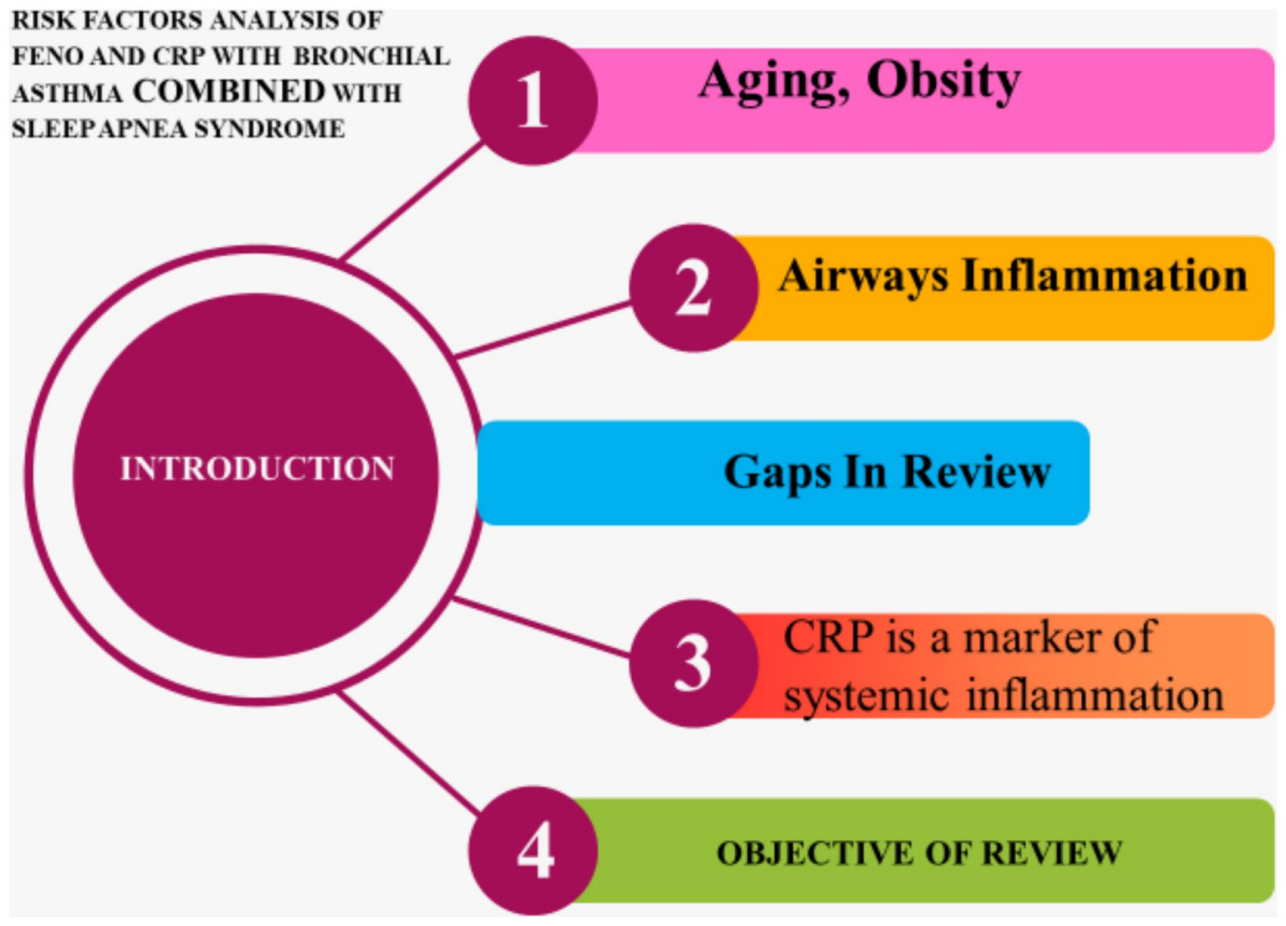
Analysis of BAS and OSA.

Both FeNO and CRP are very widely known biomarkers of inflammation. However, they are related to different aspects of the inflammatory process (4). FeNO is defined as a non-invasive measure of airway inflammation and is also a common feature of asthma. It supports the occurrence of type 2 inflammation, which includes signs of eosinophilic airway inflammation, that are important to asthma development (5). On the other hand, CRP is a systemic marker of inflammation that can be elevated in any type of disease, such as asthma and/or sleep apnea syndrome (6). It is more general and nonspecific and represents an acute phase reaction, and may be elevated in response to stimuli such as infections, trauma, or chronic inflammation. The existence of BA and co-OSA is a relatively under-researched area, and there is still limited knowledge regarding specific interaction between these conditions. FeNO and CRP have been investigated separately to asthma and sleep apnea but a few authors describe how both can be related or linked regarding the patients with both pathologies (7). Moreover, many studies have described the role of FeNO and CRP in asthma and sleep apnea; however, few studies have investigated the interconnection between these biomarkers in asthma and sleep apnea syndrome (8). However, there has not been enough investigation into the relationship between these markers, disease severity and frequency, and treatment response in patients with the two illnesses. Such a gap in the current literature is an invitation to further investigate the relationship between FeNO and CRP levels by clinicians to diagnose, assess, and treat patients with BA and OSA (Figure 1).

### The rising prevalence of bronchial asthma and sleep apnea syndrome

Global data indicate that asthma is a prevalent chronic respiratory disease that affects approximately 300 million people (8). It is defined by persistent airways inflammation and manifests through wheezing, coughing, difficulty breathing, and chest constriction. Asthma attacks that can be caused by allergens, respiratory infections, and the like entail considerable morbidity, as well as mortality if not addressed. It mainly targets the lower respiratory tract, and its cardinal feature is eosinophil inflammation, which was confirmed by the depiction of FeNO levels. Asthma is a chronic disease characterized by airway hyper-responsiveness and inflammation caused by interactions between genetic susceptibility and environmental influences.

Another prevalent global respiratory disorder is sleep apnea syndrome, with obstructive sleep apnea (OSA) being the most common form. Sleep apnea prevalence is 24% for adults with mild to severe severity (9). OSA means repeated episodes of partial upper airway obstruction during sleep, chronic intermittent hypoxia, sub-optimally quality sleep, and rise of sympathetic tone. Additionally, OSA has also been associated with numerous motor highway systemic health concerns such as hypertension, cardiovascular disease, and metabolic diseases (10). Both conditions are familiar to many practitioners, although the coexistence of BA and OSA, which is known as the co-morbidity of asthma and sleep apnea syndrome, is not the subject of extensive research. It has been pointed out that patients with asthma are predisposed to developing sleep apnea (and that is why patients with sleep apnea are predisposed to developing asthma, too) (11). Some of the ways this connection happens have not yet been determined. Several nominations have been made about specific risk factors such as obesity, aging and genetic which is reasonable but their physiologic and inflammatory link still warrants researcher. Table 1 provides information on SNPs significantly associated with obesity, including SNP ID codes, effect alleles, other alleles, EAF values, Beta values, and standard errors (S.E.) Huang et al. (12).

**Table 1:** Exposure and outcome GWAS summary.

Category	GWAS ID	Trait	Year	Sample size	Control	Case	Number of SNPs	Population
Exposure	finn-b-E4_OBESITY	Obesity	2021	218,735	209,827	8,908	16,380,465	European
Outcomes	ukb-d-I9_PAD	PAD	2018	361,194	359,964	1,230	9,637,467	European
	finn-b-I9_PAD	PAD	2021	213,639	206,541	7,098	16,380,453	European
	bbj-a-144	PAD	2019	212,453	208,860	3,593	8,885,805	East Asian
	ebi-a-GCST90018670	PAD	2021	177,713	173,601	4,112	12,454,558	East Asian
	ebi-a-GCST90018890	PAD	2021	483,078	475,964	7,114	24,186,090	European

GWAS, Genome-Wide Association Studies; PAD, Peripheral Artery Disease.

SNP refers to a Single Nucleotide Polymorphism, which is a variation in a single DNA building block (nucleotide). EAF stands for Effect Allele Frequency, representing the proportion of chromosomes carrying the effect allele in a population. Beta indicates the effect size, i.e., the strength and direction of association between SNPs and traits. S.E. is the Standard Error of the Beta value. These values help in assessing the genetic associations between obesity (as a shared risk factor) and outcomes like PAD, potentially overlapping with respiratory conditions.

### The role of inflammatory biomarkers in asthma and sleep apnea

This article has associated inflammatory biomarkers to asthma and sleep apnea syndrome pathophysiology. In asthma the inflammation of the airways is a hallmark of asthma, and eosinophils usually characterize this inflammation (13). FeNO is an invasive marker for the disease, characterizing airway inflammation by eosinophils. FeNO is formed when NO is generated through the induction of the enzyme iNOS in the airway epithelium. FeNO is usually increased in patients with asthma, particularly in type 2 inflammation, and can be used to assess disease control and predict exacerbation (14). FeNO levels also act as a standard in understanding the clinical management of children, especially in determining when to start and/or increase inhaled corticosteroids.

However, CRP is a marker of systemic inflammation generated in the liver by synthesizing pro-inflammatory cytokines such as interleukin-6 (IL-6) (15). It’s an acute phase protein not specific for COPD and is elevated in conditions such as asthma and sleep apnea. Studies have demonstrated that in asthma, CRP levels increase with disease severity, with an increase in asthma exacerbations and systemic inflammation (16). The increased concentration of CRP is also measured in patients with sleep apnea where hypoxia and then reproduced oxidative stress predetermines inflammation in the whole organism and, therefore, worsening of basic illnesses, hypertension, cardiovascular diseases, and metabolic disorders (17). While a plethora of information exists on FeNO and CRP in asthma and SAs separately, comparatively limited data has been published on the association of these two indices in patients with both BA and OSA.

A key element in the pathophysiology of asthma is eosinophilic inflammation, which is closely correlated with FeNO (fractional exhaled nitric oxide) (18). Patients with asthma usually have higher levels of FeNO because of increased inflammation in the lower airways. Both lower airway inflammation associated with asthma and upper airway inflammation that results in the development of obstructive sleep apnea (OSA) are characterized by the nitrogen oxide marker FeNO. From a pathogenic perspective, FeNO plays a crucial role in the development of both asthma and obstructive sleep apnea. Because of the inflammatory signals seen in both the upper and lower airways, FeNO can act as a disease indicator and promote the course of the disease while also hastening its onset (19). In a study of 261 participants (as shown in Table 2), no significant correlations were found between FeNO levels and AHI or OAHI in OSA, asthma, OSA + asthma, or control groups. FeNO was highest in the asthma group, while AHI, OAHI, and ODI were elevated in OSA and OSA + asthma groups, indicating greater sleep-disordered breathing severity.

**Table 2:** Key parameters by group (Median [P25–P75]).

Parameter	OSA (*n* = 68)	Asthma (*n* = 42)	OSA + Asthma (*n* = 109)	Controls (*n* = 42)
FeNO (ppb)	10 [5–27]	18.5 [9–49]	15 [6–35]	14 [7–37]
AHI (events/h)	5.9 [3.9–13]	1.1 [0.8–1.5]	5.8 [2.3–10]	1.25 [0.8–2.3]
OAHI (events/h)	4.15 [3.4–9]	0.85 [0.5–1.3]	4.4 [2.2–7]	1.0 [0.3–2.1]
T90 (% TST)	5.3 [0.8–24.7]	0.9 [0–5.5]	6.0 [0.7–19]	6.9 [0.2–23]
Nadir SaO₂ (%)	84 [80.5–86]	87 [85–89]	84 [79–86]	87 [85–89]
ODI (events/h)	11.8 [8.9–17]	6.8 [5.5–8]	12.1 [8.8–18]	7.15 [4.7–9]

FeNO, fractional exhaled nitric oxide, ppb; AHI, apnea-hypopnea index, events/h; OAHI, obstructive AHI; T90, % total sleep time with SpO₂ < 90%; ODI, oxygen desaturation index, events/h; SaO₂, oxygen saturation. This table highlights that FeNO levels are highest in asthma-only patients, reflecting strong eosinophilic airway inflammation. However, AHI, OAHI, and ODI values—markers of sleep apnea severity—are notably elevated in both the OSA and OSA + Asthma groups. These results suggest that individuals with both conditions suffer from a dual burden of inflammation and breathing disturbance during sleep, which may complicate diagnosis and management.

Further research is necessary to determine how FeNO contributes to the symptoms of asthma and obstructive sleep apnea, as this investigation may lead to the development of new diagnostic techniques and treatment strategies. It has been established that inflammation in asthma affects primarily the airways while in OSA affects every part of the body. It has been postulated, and in this study, the authors hypothesize that the additive or synergistic effect of both types of inflammation, were they to coexist, would manifest more dire clinical outcomes concerning disease flare and systemic comorbid affiliations (20).

### Gaps in the literature

Indeed, as stated earlier, several studies have been conducted on asthmatic patients and sleep apnea and their biomarkers, FeNO and CRP, separately (21). However, no comprehensive study has, to date, assessed whether the biomarkers are interrelated for patients diagnosed with both diseases. To the best of my knowledge, few studies have evaluated the relationship between FeNO and CRP when asthma is accompanied by OSA. While asthma and sleep apnea affect a significant proportion of the adult population, we found that the natural history of these diseases and whether they are linked significantly when present together are not well-defined. The limited number of studies investigating this intersection predominantly concentrates on obesity as a common underlying factor. However, more work should be done to understand the inflammatory pathways in patients diagnosed with both diseases. Further investigations are required to understand the relationship between these two conditions at the immunological level.

Most investigations of FeNO and CRP have been conducted using a cross-sectional design, and therefore, knowledge of the longitudinal correlations of these indicators with disease outcome is still somewhat limited (22). In the case of BA and OSA, future cross-sectional investigations could ascertain the relationship between changes in FeNO and CRP and exacerbations, ailment comorbidities, and treatment outcomes. The interaction between FeNO and CRP and the presence of both BA and OSA is not well explained mechanically. Although, it has been explained, both markers indicate inflammation, there are inconsistencies in how these two relate to each other in patients with both conditions. Therefore, there is a sense in studying how it is possible to modify these biomarkers, how they are interrelated when comorbid BA and OSA, and how they can affect the severity of the diseases. It still remains not clear for the clinical use of detecting FeNO and CRP in OSA patients. FeNO is used as a tool for tailor-made asthma management, and CRP is a marker of systemic inflammation. However, their application in patients with both diseases has been investigated insufficiently. Further research should focus on investigating whether simultaneous measurement of FeNO and CRP would present a better picture in managing chronic disease, help decide when to adjust treatment or predict a relapse.

### Objective of review

The present review offers an appropriate assessment of FeNO and CRP in patients with bronchial asthma accompanied by OSA. As stated in the aforementioned gaps, this article attempts to fill these gaps concerning the existing literature and formally recommend future research directions in this field. Knowledge of how FeNO and CRP work together in BA as well as OSA patients will be vital in enhancing the dexterity of diagnosing the condition, managing the disease progress, and developing treatment options that are beneficial for patients with the two diseases.

## Overview of FeNO and CRP

### Fractional exhaled nitric oxide

A significant index that is obtained noninvasively and describes airway inflammation, especially the eosinophilic inflammation underlying most respiratory diseases, including asthma. Nitric oxide (NO) is a short-lived free radical chemical compound produced directly in the human body by the action of L-arginine-pulmonary endothelial cell nitric oxide synthase. A major source of FeNO in an asthmatic environment has also been established to be derived from the epithelial cells of airways due to eosinophilic inflammation (23). It is used as a mark of the type 2 inflammation, which is crucial to the development of asthma. This biomarker has been the subject of many studies in view of its usefulness in assessing the severity of asthma, inflammation level, and the onset of asters in patients with persistent asthma. While FeNO50 values > 50 ppb in adults (or >35 ppb in children) are likely to indicate eosinophilic airway inflammation, values <25 ppb in adults (or <20 ppb in children) are regarded as normal. In the clinical setting, values ranging from 25 to 50 ppb (20 to 35 ppb in infants) should be taken with caution (Table 3). This table summarizes the ATS clinical thresholds for interpreting FeNO levels. Values above 50 ppb suggest active eosinophilic airway inflammation, commonly seen in uncontrolled asthma. These guidelines are especially useful for identifying inflammation in asthma and comorbid conditions like OSA, guiding decisions on corticosteroid therapy and monitoring disease control.

**Table 3:** FeNO50 levels and assessment of the airway’s inflammation according to the ATS guidelines (23).

FeNO_50_ < 25 ppb (<20 ppb in children)	FeNO_50_ 25–50 ppb (20–35 ppb in Children)	FeNO_50_ > 50 ppb (>35 ppb in Children)
Eosinophilic airway inflammation unlikely	Be cautious and monitor changes in FeNO_50_ over time	Eosinophilic airway inflammation present

### Biological mechanism of FeNO production

High FeNO is produced when inducible nitric oxide synthase (iNOS) is synthesized due to inflammation mediators. The role of NO in the respiratory system has duality; that is, it not only has a beneficial effect on health but is also involved in harm to health. On the one hand, NO helps prevent the running of tone, and the beserk controlling bronchodilation, which is a physiologic constant in the lungs. In contrast, elevated FeNO in asthma reflects eosinophils, which release toxic potent inflammatory cytokines such as IL-4, IL-5, and IL-13 that induce airway alterations termed sensitization and swelling (24). FeNO and exhaled NO have been understood as a marker of eosinophil activity in the airways and with regard to the severity and intensity of asthma, and the airways’ sensitivity (25). An LMN FeNO is a feature of atopic asthma and the resultant pathology is attributed to IgE mediated inflammation (26). This biomarker is also beneficial for measuring treatment effectiveness because FeNO levels usually drop after the best anti-inflammatory management, including ICS.

### FeNO clinical practice

FeNO plays several roles in asthma management.

It also provides useful info on the extent of anti-inflammatory treatment in patients with poorly controlled asthma, the FeNO levels are high (27). More over FeNO can be used as a therapeutic weapon for titration in combination with ICS in individually: according to the patient inflammatory activity level. Higher FeNO levels in patients with asthma are associated with an increased likelihood of an asthmatic attack. Therefore, FeNO can be used as the prospective biomarker of ER visits or hospitalization due to asthma, possible in the nearest future (28). A typical FeNO in patients with prescribed corticosteroids might point toward non adherence to medication since corticosteroids break down FeNO levels (29). It seems that FeNO is very useful in the assessment and evaluation of the eosinophilic asthma patients who require corticosteroids. Moreover, FeNO levels are directly associated with the degree of airflow obstruction in patient with COPD as well as other related respiratory diseases (30).

### Limitations and challenges in using FeNO

**Figure 2:**
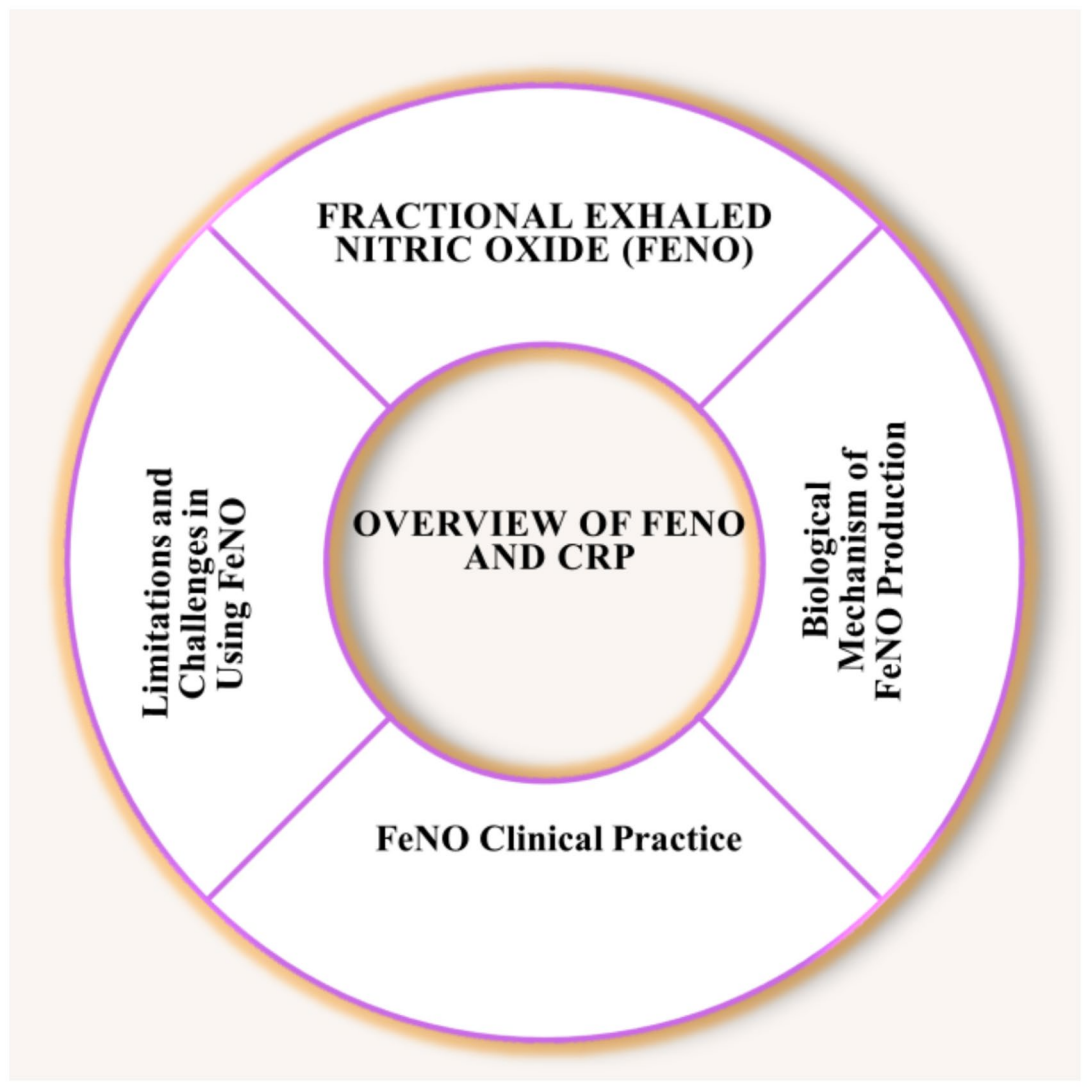
View of FeNO and CRP.

FeNO has proven valuable in both epidemiological and clinical research. Nevertheless, its measurements are subject to various influencing factors such as age, gender, smoking history, and co-morbidities, including chronic rhinosinusitis (CRS) (31). Additionally, FeNO is limited in its ability to assess inflammation across all asthma phenotypes, particularly in neutrophilic and mixed-type asthma (32). Therefore, FeNO should be considered a supplementary marker in the diagnosis and treatment of SPD, rather than serving as the primary indicator (Figure 2).

### C-reactive protein

CRP is an apolipoprotein that is synthesized in the liver in inflammation and is mainly related to general inflammation. It is raised because of inflammatory cytokines especially IL-6 and they are increased during infection, trauma or in chronic inflammatory disease (33).

### Biological mechanism of CRP production

CRP is synthesized by the liver using cytokines especially the interleukin-6 during episodes of inflammation. Therefore, under inflammatory conditions, IL-6 activates JAK/STAT signaling, which results in the transcription of the CRP gene in hepatocytes. It appears that C-reactive protein is released into the blood stream in this process (34). It then binds to phosphocholine found on the surface of apoptotic cells and bacteria, and initiate the complement process whereby apoptotic body are eliminated from the body (35). Regarding asthma, sleep apnea, and other chronic diseases, the rise in CRP levels is attributed to chronic, low-grade inflammation that fosters pathophysiological processes and comorbidities.

### CRP in asthma

The main characteristic of asthma lies in eosinophilic inflammation yet this inflammatory process usually does not result in increased C-reactive protein (CRP) levels, but airway obstruction from OSA discourse may explain the CRP elevation (36). Patients with OSA often present with systemic inflammatory responses which raise CRP amounts although this increase stems indirectly from combined pathophysiological influences of OSA together with asthma (37). Doctors should interpret increased C-reactive protein results in people with OSA and asthma as indicators of OSA-related inflammation because this elevation does not stem from asthma conditions. The correct comprehension of this distinction helps explain the intricate link between these two medical disorders.

In asthma their CRP values normal to slightly raised, but not increase like those of their counterparts when have infection or inflammation. But in cases of relapses or in patients with more severe disease patterns, it can notably raise—up to 10 mg/L As the data shows, elevated CRP levels are reported as relating to more extensive asthmatic signs and high levels of airway inflammation (38). Moreover, it has been found that elevated levels of CRP are associated with vascular inflammation and cardiovascular morbidity in asthma because people who have severe asthma are most likely to suffer from other complications like heart diseases, etc. Airway remodeling is considered an integral feature of asthma and is characterized by wall thickening, smooth muscle hypertrophy in the airway walls, and increased extracellular matrix deposition (39). These changes are because progressive inflammation is partly expressed in the level of CRP (40). Other aspects of inflammation like CRP that also rises in asthma is directly associated with airflow resistance and diminished lung functionality in order to grade the asthmatic condition (2).

### CRP in combined asthma and sleep apnea

**Figure 3:**
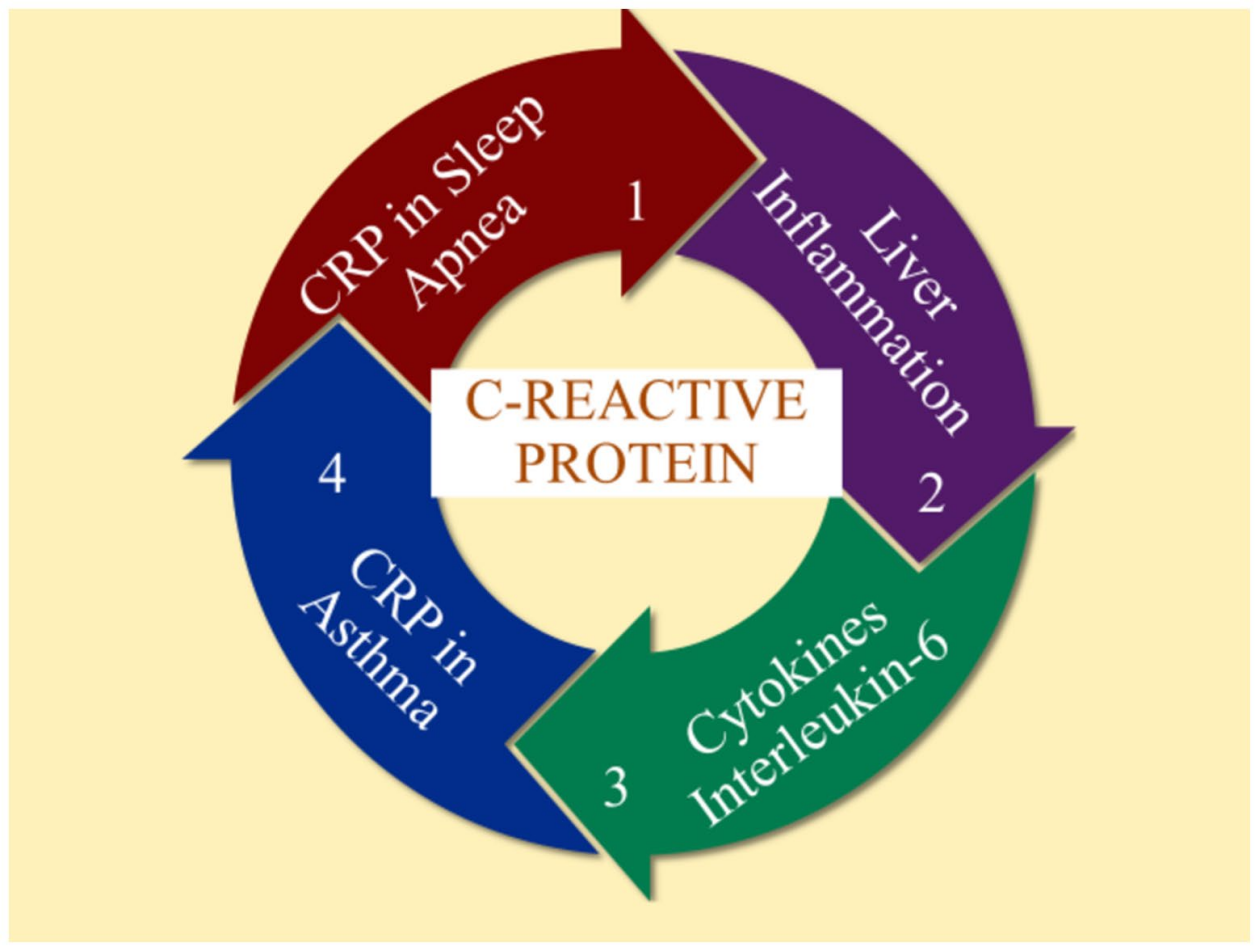
C-reactive protein synthesis and mode of action.

Diseases associated with asthma are multifaceted, especially because obstructive sleep apnea has an inflammatory effect on the level of systemic inflammation (41). Elevated mean CRP levels have been reported in those patients with both asthma and sleep apnea than in subjects with only sleep apnea (42). This might be because of the fact that irritation of the air passage in asthmatic patients is associated with sleep apnea, which results in systemic inflammation. These two conditions may have a presenting syndrome, where worsening of either precipitates or worsens the other and leads to a poor prognosis for both diseases. Hype in CRP in these patients signifies not only an extent of airway inflammation but also evidence of co-morbid conditions such as hypertension, cardiovascular disease and obesity. COPD contains inflammation signs such as asthmatic airway which can reemphasize its chronicity and threaten patient survival, while sleep apnea is a pattern of systemic inflammation that also sustains or hazardize the chronic condition each whether (Figure 3).

## Association between FeNO, CRP, and bronchial asthma combined with sleep apnea syndrome

### Comorbidity of asthma and sleep apnea syndrome

Two obstructive respiratory diseases, severe bronchial asthma and sleep apnea syndrome, when comorbid, cause a great number of problems for the patients. Asthma, being an inflammatory disease of the airways that involves hyperresponsiveness of airways, inflammation of mucosa and airway obstruction (43). Obstructive sleep apnea (OSA) is defined by the presence of multiple partial or complete upper airway obstructions during sleep, resulting in normal or diminished ventilation, hypoxia and inflammation (44). Coexistence of asthma and sleep apnea not only worsen each other’s clinical manifestations, but also dysregulate the patients’ clinical indicators. OSA and asthma exhibit participatory inflammatory processes; in particular, the role of type 2 inflammation in asthma and systemic inflammation in OSA. Fractional exhaled nitric oxide (FeNO) and C-reactive protein (CRP) are biomarkers that define the level of inflammation in these diseases. Thereby, both markers express information about the courses of asthma and effects of sleep apnea on the systemic level. In what follows we will focus on physio pathological correlations of FeNO and CRP with asthma and sleep apnea. The interactions between both conditions will also be discussed concerning either of them, the combination of both, diagnosis, management, and treatment implications.

### Role of FeNO in asthma and sleep apnea

FeNO is a good indicator of eosinophilic airway inflammation which is the main characteristic of allergic asthma. It measures airway nitric oxide which is higher during inflammation, and especially during type II inflammation that is Th2 cytokines such as the IL-4, IL-5 and IL-13 (45). The FeNO increases with the degree of eosinophilic inflammation in asthma, making it a valuable diagnostic and monitoring asset. In asthmatic patients, FeNO values give a clue regarding asthma control and inflammation profusion (46). In contrast, FeNO has a diverse function in obstructive sleep apnea (OSA), especially obstructive sleep apnea (OSA). OSA results in hypoxic episodes which in turn generate oxidative stress and initiate inflammatory pathways all over the body (47). Some research has postulated that FeNO may be higher in OSA, especially given the increased expression of nitric oxide synthase, arising from hypoxic bursts, though such results are still contingent (48). Furthermore, the increase of the systemic inflammatory response due to having OSA may predispose the lower airway to increasing airway sensitivity and thereby worsening asthma symptoms (49). This indicates that FeNO can potentially use to address the goals of biomarker for both asthma airway inflammation and OSA systemic impact.

Kiaer et al. (21) found that FeNO levels may be significantly elevated in patients with comorbid asthma and sleep apnea. The airway inflammation in asthma along with the systemic inflammation in sleep apnea could add up to an increased inflammatory load in the airways which could result is serious exacerbations. Higher FeNO can therefore predict more intensive handling on both diseases (27). Recent studies highlight distinct CRP patterns in comorbid asthma-SAS. For example, Zhou et al. (50) reported significantly higher CRP levels in severe OSA patients (AHI ≥ 30) compared to controls (4.2 mg/L vs. 1.5 mg/L, *p* < 0.01). In asthma, uncontrolled cases (ACT score <15) showed CRP elevations >3 mg/L (OR: 2.1; 95% CI: 1.4–3.2) (27), suggesting systemic inflammation is driven more by OSA than asthma alone.

### Role of CRP in asthma and sleep apnea syndrome

C-reactive protein (CRP) is an acute phase protein, synthesized in the liver in response to an inflammatory stimulus. Some of these are solely active by the pro-inflammatory cytokines with IL-6 being a key stimulant of its synthesis (51). High levels of CRP show that inflammation exists throughout the body and the high levels of CRP are associated with different diseases such as cardiovascular diseases autoimmune diseases and respiratory diseases. In asthma, the level of CRP is proved to be useful in tracking the scope of systemic inflammation. Although asthma is mainly a disease of the respiratory tract, it has recently been characterized as a systemic disease. Prolonged inflammation of the airways may affect vessels with an increased risk of cardiovascular morbidity affecting just the lungs (52). It has been established that CRP levels are high in asthmatic patients and such patients have more uncontrolled asthma, frequent exacerbations and other complications (53). Moreover, CRP has also been indicated to have positive association with airway changes and poor lung function in asthma patients which make it a good indicator of asthma progression (54).

As for sleep apnea syndrome and more specifically OSA, CRP levels are still raised and are the result of the systemic inflammation promoted by IH. OSA is consistently associated with periods of airway blockade during sleep resulting in hypoxia, increase in oxidative stress and inflammation of certain pathways (55). The condition in OSA that causes most tissue hypoxia is known as intermittent hypoxia and results to the secretion of pro-inflammatory cytokines like IL-6 that in turn promotes the production of CRP (56). Therefore, OSA patients have significantly higher median of CRP than normal showing the systemic nature of the disease (50). This means that general CRP readings could be much higher in patients with both asthma and sleep apnea than in those with only one or the other disease. There is a possibility that there may be a synergistic effect between asthma inflammation of the airway and systemic inflammation from OSA to increase CRP as a marker of disease severity. The CRP has also shown to be higher in asthmatics and patients with sleep apnea, and their prognosis has been predicted by higher CRP levels (57).

### Interactions between FeNO, CRP, and comorbid asthma and sleep apnea syndrome

FeNO is single one of the most reasonable biomarkers for evaluating the eosinophilic airway inflammation in asthma as a disease which may be worse by sleep apnea. Interruptive hypoxias caused by Obstructive sleep apnea could lead to further irritation within the airways, and even airway sensitization (58). These patients have increased FeNO levels, which appear to be effective in assessing asthma control and predicting future asthmatic episodes. Moreover, FeNO can be used as a tool for the evaluation of treatment efficacy, especially for inhaled corticosteroids that are used in asthma treatment and in part for the inflammation that occurs due to sleep apnea (59).

CRP as a marker for systemic inflammation explain increased levels of CRP in patients with both asthma and sleep apnea as the consequence of systematic inflammation. CRP level is higher in those patients with both conditions as compared to patients with asthma alone that may imply that the systemic inflammation associated with sleep apnea can exacerbate the inflammatory state of asthma (60). Fewer studies correlated elevated CRP with increased vascular inflammation, cardiovascular events, and worse asthma control, suggesting that CRP-based assessment of the global disease burden in patients with comorbid asthma and OSA is feasible.

## Clinical implications of FeNO and CRP in comorbid asthma and sleep apnea syndrome

Understanding the relationship between FeNO, CRP, and the comorbidity of asthma and sleep apnea has significant clinical implications.

### Diagnostic value

In addition, FeNO and CRP are useful to classify the severity and inflammatory component of asthma and sleep apnea in clinic. It is higher in patients with poor asthma control and may increase in children with elevated FeNO levels. It has also been established that the use of a combination of biomarkers could enable clinicians to screen for the severe asthmatic at risk of repeat severe exacerbations, cardiovascular events or poor prognosis (61).

### Treatment monitoring

FeNO levels can be incorporated into the evaluation of asthma control as it relates to the inhaled corticosteroids and leukotriene antagonists. Assessment of FeNO can help in understanding the extent to which asthma inflammation in patients is being controlled, especially among patients with comorbid conditions, such as sleep apnea. Both can be used to inform treatment plan regarding co-existing asthma and sleep apnea (62).

### Cardiovascular risk stratification

This research shows that asthmatic patients who SNORING found to have high level of CRP raises cardiovascular disease risk. Both airway and systemic inflammation have been found to increase the risk of vascular injury, and elevation of their biomarkers such as AM and CRP may aid in identification of patients at increased cardiovascular risk that need screening (63).

### Tailored interventions

A useful therapeutic implication of this study would be to understand how FeNO and CRP levels correlate or differ in asthmatic and sleep apnea patients. For instance, several studies reported reduced positive effects of continuous positive airway pressure (CPAP) therapy for OSA and decreased concentration of CRP, which can control asthma in patients with OSA (64). Similarly, improving the treatment of asthma to minimize inflammation of the airways may contribute to less general inflammation that patients with both diseases experience. Combined biomarker analysis reveals synergistic effects: comorbid patients with FeNO ≥35 ppb and CRP ≥ 3 mg/L had 3.5-fold higher exacerbation risk (95% CI: 2.1–5.8) than those with normal levels (21). CPAP therapy reduced CRP by 1.8 mg/L (*p* = 0.003) in OSA-asthma patients, underscoring its anti-inflammatory role (65).

## Clinical implications and management of FeNO and CRP in bronchial asthma and sleep apnea syndrome comorbidity

Bronchial asthma associated with obstructive sleep apnea (OSA) act as a severity and progression of the two disease based on the shared pathophysiology. Asthma, which is defined by airway inflammation and bronchial hyperresponsiveness, and obstructive sleep apnea (OSA), which is defined by recurrent partial or complete airflow obstruction and systemic inflammation, are both inflammatory conditions that possess significant risk factors in common with each other including obesity, smoking history and heritability. In this context, FeNO (fractional exhaled nitric oxide) and CRP are the two essential biomarkers to study the inflammatory processes of these diseases. FeNO is a sign of eosinophilic airway inflammation that is characteristic of asthma, while CRP is an inflammatory general biomarker that is increased in both asthma and SDB (66). The awareness of these biomarkers and its clinical significance in patient management including asthma and obstructive sleep apnea is important in order to enhance prognosis.

This section will be devoted to discussing effectiveness and applicability of FeNO and CRP for clinical practice of patients with asthma and OSA. We will discuss how these biomarkers help in diagnosis, treatment, observing the course of the diseases, and treating possible comorbidity related to both of them.

### FeNO as a biomarker in comorbid asthma and sleep apnea

FeNO is a valid biomarker that has been previously applied in asthma to quantify airway inflammation of eosinophilic origin. Nitric oxide is produced by nitric oxide synthase (NOS) and is increased during inflammation of the airways especially with type 2 inflammation through Th2 cytokines (67). Increased FeNO values reflect asthma severities, poor asthma control, and eosinophilic inflammation making the test relevant in asthma management (23). However, its application in sleep apnea cases incurs ambiguity in cases of FeNO. Obstructive sleep apnea (OSA) cause intermittent hypoxia, a condition that results in increased inflammation and oxidation in the body (19). Mentioned that OSA can affect FeNO levels, may be due to upregulated nitric oxide synthase due to intermittent hypoxia (IH). In addition, OSA also induces inflammation on the upper airway and may also affect the lower airway, including asthmatic patients and contributing to elevated FeNO levels (68).

Symptoms of both asthma and sleep apnea may also lead to high FeNO levels because of inflammation from both diseases. Hos forcing asthma and systemic inflammation OSA, FeNO levels may go up, and this is a sign that requires more treatment intensity. FeNO offers clinicians information and data to manage asthma and confirms that patients with comorbidities of other diseases receive the appropriate treatment.

### FeNO in asthma treatment monitoring

FeNO is employed to evaluate the outcome of treatment in patients with asthma (27). More specifically, it can assist in optimizing the therapy with the use of inhaled corticosteroids which are considered to be the mainstay of asthma drugs. When airway inflammation is present in patients who have been administered ICS, FeNO levels would be observed to reduce (25). The clinicians perform tests known as FeNO to track the extent to which asthma therapy is effective or if the medication should be altered. In patients with asthma and coexisting sleep apnea, FeNO can generate more information about how sleep apnea inflammation may affect the severity of asthma. This is especially helpful since OSA-induced inflammation may worsen asthma symptoms, so that tracking FeNO in the patient provides both airway and systemic inflammation.

### FeNO and CPAP therapy

Continual positive airway pressure (CPAP) is also a highly recommended therapy used in treating Obstructive Sleep Apnea. As suggested by the acronym, the CPAP prompt maintains a given pressure in the airway throughout the sleeping duration to negate the possibility of the airway collapsing (69). Other benefits of CPAP treatment include the decrease in inflammation across the entire body in OSA patients, offered by lower CRP levels (65). Through its anti-inflammatory action, it is proposed that CPAP might also indirectly act on asthma and reduce FeNO levels on patients with comorbid asthma and OSA.

### CRP as a marker in asthma and sleep apnea

C-reactive protein (CRP) is another acute phase protein which belongs to the systemic inflammation and is produced in the liver due to stimulating influence of pro-inflammatory cytokines including IL-6. It is employed to evaluate intensity of the condition in the body and is used in disorders such as asthma and sleep apnea. Studies have reported that CRP is elevated in asthmatics, and is related to poor asthma control, frequent exacerbations and systemic inflammation. In OSA, CRP levels are raised by a continual fluctuation in oxygen levels which triggers the production of cytokines that in turn stimulate CRP production (70).

### CRP in asthma management

In patients with asthma, CRP is a marker of the extent of systemic inflammation, if present. Increased CRP levels are associated with increased asthma severity, higher frequency of exacerbations, and airway changes that lead to reduced lung function (71). Furthermore, although it is not exclusive to asthma, CRP is a valuable additional biomarker to determine the global inflammatory response and adjust therapy in conditions where the general inflammation is involved (72). Where symptoms of asthma are compounded by sleep apnea, CRP can therefore offer supplementary details about objective inflammation. Intermittent hypoxia enhances a low-grade systemic inflammation and thus contributes to increased airway reactivity and impaired lung function in asthma patients with sleep apnea. These patients can be regarded as having potentially more severe inflammatory processes, both in the airways and in the general circulation, and thus require more intensive treatment of asthma to achieve CRP reduction.

### CRP and cardiovascular risk

Hence besides measuring asthma control, CRP has been known for quite sometimes to be a cardiovascular disease risk indicator. Chronic diseases such as asthma and sleep apnea are also associated with cardiovascular diseases which include hypertension Coronary artery disease and stroke (73). Since both conditions are associated with systemic inflammation, this will predispose the patients to vascular damage and therefore cardiovascular events. High CRP levels were observed in patients suffering from comorbid asthma and sleep apnea are due to the enhanced cardiovascular risk in these patients to warrant improved cardiovascular surveillance among clinic patients.

## Clinical management of comorbid asthma and sleep apnea

In patients with asthma and OSA, all the principles of management in comorbid patients must include not only airway inflammation but also systemic inflammation. Early diagnosis, drug therapy, life style changes, and biomarkers such as FeNO and CRP should be routinely measured and monitored in these patients.

### Pharmacologic treatment

The goals of initial management for asthma are which are inhaled corticosteroids, bronchodilators and leukotriene modifiers (77). In patients with asthma and sleep apnea, treatments may have to be prescribes specifically for the condition with the understanding that sleep apnea will worsen the asthma. CPAP therapy is the reference treatment for OSA and has been reported to decrease markers of systemic inflammation in patients with the disease (74). Thus, in addition to asthma medications, the use of CPAP therapy controls both airway and systemic inflammation and may enhance asthma control by decreasing FeNO and CRP. If a patient has severe asthma or did not benefit from ICS medications, further treatments including biological agents which act on IL-5 (mepolizumab, reslizumab), IL-4/IL-13 (dupilumab) or IgE (omalizumab) can be used. These therapies are aimed directly at the type 2 inflammation implicated in both asthma and allergy, may offer better control in those with co-morbid sleep apnea.

### Lifestyle modifications

Diet and exercise are critical in asthmatic patients, and any measures that improve sleep are significant in sleep apnea. Intervention such as weight loss causes an improvement in all forms of sleep apnea more so in obesity related sleep apnea (75, 76). Also, constitutional measures like smoking cessation, avoiding known allergens, and air borne irritants, are known to help decrease both local inflammation in the airways and systemic inflammation.

### Monitoring and follow-up

CRP and FeNO are useful parameters for assessing inflammation of both asthma and sleep apnea, their levels should be monitored on a coherent basis. FeNO is a marker for persistent eosinophilic airway inflammation, with a normal range of 25–150 parts per billion; elevated CRP defines the condition with a normal range of 0–8 mg/L (23). Serum biomarkers, when measured at routine follow up visits, can help the clinician evaluate treatment outcomes, modify therapy when necessary, and risk stratify patients for Adverse Asthma Outcomes or Acute Cardiovascular events.

## Conclusion

Asthma-OSA overlap is a clinical issue requiring a multifaceted approach to diagnosis and management. Understanding these two conditions is crucial for patient care. Biomarkers like FeNO and CRP measure inflammation in asthma and sleep apnea comorbidity, determining airway inflammation and eosinophilic response. Higher FeNO levels are associated with asthma worsening, inadequate asthma control, and increased airway irritability. Sleep apnea can elevate FeNO due to both asthma and systemic inflammation. Intermittent hypoxia, followed by increased systemic inflammation, worsens asthma symptoms, increasing FeNO levels. Therefore, FeNO in patients with both asthma and nasal polyposis provides crucial information about airway inflammation, aiding in adjusting management plans for both diseases.

CRP is a biomarker linked to airway inflammation, whereas FeNO is linked to airway inflammation. It is synthesized in the liver during inflammation and released by cytokines like IL-6. Increased CRP is found in both asthma and sleep apnea, with SH having a direct positive correlation with CRP. CRP levels above 5 mg/L are associated with asthma flares, poor asthma control, and higher cardiovascular events. When combined, both local airway inflammation and systemic inflammation from sleep apnea must be treated. Non-pharmacologic management includes bronchodilators like ICS for asthma and CPAP for sleep apnea, which can relieve airway inflammation in asthma and decrease IH and systemic inflammation in sleep apnea patients. Biologic agents targeting specific inflammatory mediators can be used for severe asthma patients.

Adherence to medication and lifestyle modifications are recommended for asthma and sleep apnea treatment. Weight loss can reduce severity of sleep apnea and asthma control. To reduce inflammation, patients should avoid triggers such as allergens, air pollutants, and cigarette smoking. Lifestyle changes like diet, exercise, cessation of smoking, and weight management can help reduce inflammation and relieve asthma. Biomarkers like FeNO and CRP should be routinely checked in patients with concurrent asthma and sleep apnea to assess disease outcomes, compare therapeutic approaches, and identify patients with increased risk factors. These biomarkers help make data-based decisions about therapy choices.

Patients with asthma and sleep apnea have higher systemic inflammation and limited circulation, making them at higher risk for cardiovascular disease. High CRP levels indicate poor high-density lipoprotein cholesterol and hypertension, which are often identified in these patients. Cardiovascular health monitoring is crucial in this population group, as blood pressure checks, lipid profile, and cardiac tests can reveal early signs of cardiovascular diseases and inform preventive strategies. Examining FeNO and CRP levels helps clinicians understand the intricate interplay between asthma and sleep apnea, making asthma more manageable, reducing exacerbations, and minimizing the impact of sleep apnea on patients. By utilizing these biomarkers and other clinical tools, clinicians can gauge the risk of cardiovascular disease development, assess treatment effectiveness, and achieve better patient outcomes. As research advances, FeNO and CRP will continue to demonstrate increasing clinical utility in managing this complex patient population.
